# The Diagnosis and Treatment of Tubercular Peritonitis*Read before the Tri-State Medical Association of Virginia and the Carolinas, in session at Charlotte, N. C., Jan, 11-13, 1899.

**Published:** 1899-03

**Authors:** Wm. L. Robinson

**Affiliations:** Danville, Va.; Ex-President Medical Society of Virginia, Etc.


### THE DIAGNOSIS AND TREATMENT OF TUBERCULAR PERITONITIS.*

BY WM. L. ROBINSON, M.D., DANVILLE, VA.

Ex-President Medical Society of Virginia, Etc.

The subject of tubercular peritonitis is one full of interest both to the general practitioner and surgeon. The disease for years was regarded as incurable, till surgery opened up a field promising much, which has been realized in certain forms. More recently, while no special curative agencies in the therapeutical line offer anything of significance, statistics show from fifteen to thirty per cent. of spontaneous cures, especially in the young. Reyburn and Hughes Bennett state that from examination of cadavers, from one-third to one-half of all individuals who die after the age of forty have had spontaneous arrest of tubercle in some stage.

While all forms of tubercular peritonitis are full of interest, I desire more especially to allude to some points pertaining to the more acute form.

Whatever division be made, whether of fibrous, ascetic, seromembranous, purulent or ulcerative, there are two leading features in tubercular peritonitis—one is plastic exudate; the other effusion. The first is always present; the latter, absent in many cases.

The varied symptomatology of invasion and progress forces diagnosis by careful exclusion.

1. In one, you will find it marked by insidious, slow and even advance, without or with only occasional pyrexia, and with ascites as an early and leading trait.

2. Again, we have a series of attacks, with intervening lulls, until the entire peritoneal cavity has been attacked, without effusion, but retraction of the abdomen.

3. Again, chills, with fever, or seemingly invasion of typhoid fever, marks its progress.

4. The sudden invasion after miscarriage.

5. Gradual increase of swelling, resulting in enlarged abdomen, with more or less pain (abdominal), and pain in urinating.

6. Again, effusion may begin without abdominal tenderness, without a febrile condition, and with nutrition unaffected. Such a condition, unexplained in some other way, should arouse suspicion of tuberculosis.

When you can trace the invasion from the pelvis above, with floating masses in the abdomen, and deep intestinal percussion sound, the diagnosis is usually clear.

I have noticed, in manipulating the abdomen with hand extended, under the finger-bulbs, on deep pressure, a crepi-

*Read before the Tri-State Medical Association of Virginia and the Carolinas, in session at Charlotte, N. C., Jan. 11-13, 1899.

tant feel, like the sensation of air or water infiltration in cellular tissue. Again, I have observed under such pressure the gurgling of displaced gas, as you find in adhesive appendicitis.

The history of the case, with the impression the individual case makes on you (like deciding when to operate for appendicitis), enables you to sum up a diagnosis; yet you can not formulate in words or rules the guide thereto.

I can add something, possibly, to these diagnostic hints, by briefly reviewing the history of several cases. First, I would report three cases of young children who came under my care in the last twelve months. One was rosy and stout, of tubercular ancestry; the other two were of average robustness,

CASE I.—Mr. D.'s child, aged six years, had several chills, followed by fever, and then a regular evening rise of temperature to 103° for ten days, and then an intermission of a week, with fever recurring thereafter irregular in its history; then exacerbations, often the highest in the morning, but abating several times in twenty-four hours; constipation persisted in spite of several mercurial purgatives. There were abdominal pains, with irritable bladder; the appetite was a marked feature, never failing under fever or quinine, the latter faithfully tested for the first ten days, sponging, hot douches over abdomen, painting with iodine and collodion over bowels, sun-baths, massage, etc. Stay of two months in the country gradually dissipated the symptoms, and now six months elapsed and health perfect.

CASE II.—Miss M., aged nine years, tubercular ancestry, commencing indigestion pains in abdomen, constipation, slight fever, increasing in evening, becoming irregular, crepitant feel and gurgling of gas under pressure, pulse rapid, hectic flush occurring several times daily on first cheek and then on other, later complicated with pleuritis. Treatment: Saline irrigation, iodine over abdomen, and massage, fresh air, and special care of digestion and building up, with apparent restoration of health. There was fluid felt in flanks when examined with attention to position; sick two and one-half months; recovery.

CASE III.—Mr. W. W.'s child, aged six years, rosy and active; invasion gradual, pain in bowels a few days, slight fever, malaise, but appetite good for three months. This condition of slight attack and lull alternated, till recently an invasion simulating typhoid fever came on, with tenderness persistent over abdomen; temperature normal in the morning, and 101° or 102° F. in the evening. Mercurial purgatives and quinine availed nothing, and promptly discontinued; later the abdominal tenderness became localized in small areas. The crepitant sensation was marked in this case; later the tenderness disappeared, but promptly recurred on walking around; the appetite was excellent and urgent all the while. She is still under observation. Some light will be thrown on this case by this history of the next one, who was her sister.

CASE IV.—Miss M. W., aged seventeen years, tuberculous history; pelvic tenderness for three years, frequently suffering violently, especially at menstrual period; she was always excessive in flow, and too frequent. Out-door exercise, and even the bicycle, seemed to improve her every symptom and general health. I advised operation two years ago, recognizing the tubercular involvement of the appendages. The last attack simulated typhoid fever, ran a history of great violence, nausea, abdominal tenderness, prostration, insomnia, irregular fever from sub-normal to 106°. I tided her over the first two weeks, and she was on her feet, with fine appetite. The recurrence was attended with constant fever of 104 to 106, and after five days of inability to nourish, and fluid forming, I did a laporotomy; temperature subsided promptly, but died from exhaustion; the appendages, pelvic and surrounding tissues were studded with tubercles.

I omitted to state that the six-year old sister menstruated two months before last attack.

CASE V.—Dr. Spencer's case: Young lady, fifteen years of age, had been suffering with general tenderness and apparent appendicitis, with pain and fever, for months. Operation for appendicitis. The whole of the peritoneum and intestines were matted together, modulated and studded with tubercles as thick as possible. The whole intestines were agglutinated in one mass, and scarlet red. The adhesions were liberally broken up, appendix removed, flushed and wiped dry, dusted with iodoform, and walled off with gauze. Recovery rapid.

CASE VI.—Mrs. Hindly, aged 32. Referred to me by Dr. Smith, of Henry. Temperature for eight weeks had ranged from 97 in the morning to 104½ in evening. Abdomen distended, and ascitic; diagnosis easy; operation revealed floating gelatinous masses filling the abdomen and peritoneum, and all inner surfaces studded with tubercles. Irrigated, dried with gauze, dusted freely with iodoform, and walled extensively with gauze; temperature did not reach 100 after operation; gained twenty pounds in five weeks; lived six years, bore a healthy child and died of pneumonia.

CASE VII.—Miss S. T., aged eighteen; always regarded as a healthy girl; had been treated for typhoid fever for seven weeks; morning temperature subnormal, afternoon 103½ or more; fine appetite; going around. The above was the history when called in. She was a niece of the last-named lady, and I diagnosed tubercular peritonitis; operated and found adhesions too strong to be broken up. Liberated as far practicable. Temperature remained normal for seven days, and apparent improvement, then recurrence of trouble, ending fatally in eight weeks. She was a mere skeleton when I saw her. I am inclined to think had operation been performed when adhesions were slight a different result might have been attained.

### TREATMENT.

It is stated that from fifteen to thirty per cent. of acute cases spontaneously recover. It is significant that the tendency of the disease to self-limitation is sufficiently marked to cause so careful an observer as Kaulich to define it by a separate group in his classification of the different forms. This tendency is not confined to tuberculosis of the peritoneum, but here, rather than elsewhere, the result is more possible of attainment.

The lungs appear to be the region in which the life-history of the bacillus attains its perfect fulfillment; here it multiplies with greatest activity, and as a result produces the most disastrous effects upon life and tissue. In the lungs the normal environment favors rather than retards the extension of the invasion. Yet, in a paper based upon the result of 1,146 post-mortems at Bellevue Hospital, Henry P. Loomis gives a very large percentage of observations showing spontaneous cicatrization lung lesions after tuberculosis. In the researches of Loomis, we have the logical inference before us that the ratio he observed of spontaneous cicatrization in the lungs may be assumed to be true of the peritoneum, plus the enhanced tendency of the latter part to limit the extension of disease; but to accomplish this, either in the lungs or peritoneum, we must have unimpaired a certain vital antagonism to the disease. Osler says: "There is no inherent improbability why tuberculosis of the peritoneum should not undergo involution as they do elsewhere. Anatomically, the peritoneal growth bears in its evolution a close analogy to the pulmonary; and this is further borne out by the retrograde changes through which it passes, just as the aggregations of miliary nodules on the lungs may undergo the changes we speak of as healing, becoming hard and fibroid, so in the peritoneum, the tubercle tends in many cases to become sclerotic, and passes into a condition in which it is practically harmless."

Tuberculosis being a disease of exhaustion by pyrexia, spontaneous cure must largely depend on extent of invasion, then the question resolves itself as to how we can sustain the vital forces as the resistent agent by keeping intact the assimilating powers.

There we see the largest percentage of recurrences in the young when the nutritive forces are at the summit of activity. Our efforts should be devoted to the improvement of the general health by proper attention to the digestive organs, the regulation of the bowels, and proper dietary, avoiding fermentation producers, using irrigation of colon, with saline washes, sponge baths, massage, pouring hot water over abdomen, painting abdomen with iodine. Bedford alum-water or mass, on account of the iodine and iron it contains, is indicated. Correction of constipation, sunlight and fresh air is of paramount importance.

The operative side is attractive, because we often get prompt and striking results. Not only in the ascitic form, but in the class of cases with retracted abdomen, when exudative adhesions are not too strong, I see no reason for non-interference, careful severing of adhesions, wiping out with dry gauze, free dusting with iodoform, and extensive walling off with iodoform gauze is indicated. The effect of iodoform in tubercular joints is certainly suggestive of its possible good influence in a tuberculosis of the peritoneum.

While we recognize that the results of laparotomy are strongly encouraging, yet the explanation of how it cures is still unsatisfactory, so we go on empirically. Nevertheless, I will impose on your time by reading an article by Hildebrandt on the causes of the healing influence of laparotomy in tubercular peritonitis. It is at least interesting and ingenious. He says tubercular peritonitis is undoubtedly cured by laparotomy, but satisfactory explanation is lacking, and the various theories of this puzzling fact call for serious reflection.

The writer first speaks of the appearance resulting from abdominal incision of the unchanged (healthy) peritoneum. He experimented on dogs and cats by dividing the various layers of the abdominal walls, closing the same. After some time he re-opens, and finds as a result of the laparotomy, besides the paralysis of the intestines, which is observed after every operation upon the abdomen, and which leads to meteorism and constipation, a distinct hyperæmia, which may continue for a week after the operation, which he looks upon as venous from its appearance. This hyperæmia is in part due to the faulty contraction of the bowel. The constant peristalsis is a powerful means of ridding the intestines of venous blood, and when this is absent there results a congestive hyperæmia just as in paralyzed parts in other portions of the system. The hyperæmia is also due to inflammation, which, the writer holds, occur in every aseptic laparotomy.

As the nature of the inflammation is not definitely established, the physician clings to the cardinal symptoms, and diagnosticates (inflammation). The tumor, visible at the external portions of the body, corresponds to increased exudation in the peritoneal cavity, which is found after every laparotomy upon re-opening the abdomen. A few days after the operation, there is seen in the free peritoneal cavity a slight quantity of a reddish fluid, consisting of cast-off epithelium and a few pus cells. These would be present in greater amount were it not for the enormous absorptive properties of the peritoneum.

The local production of warmth and sensitiveness is the result of increased blood-supply.

In tubercular peritonitis, there is likewise an active hyperæmia set up in consequence of the reaction upon tha peritoneum following opening of the abdomen, and seems to be even more intense than that produced upon the normal unaltered peritoneum. This is probably due to the circumstance that the inflamed tissues hold the blood more firmly in the dilated vessels.

The writer noticed that when laparotomy was done in the early stages of the disease, where no retrogressive changes have yet taken place, no effect upon the condition occurred. This fact is of importance in the explanation in the curative effect of operation. Tubercular peritonitis frequently tends to spontaneous cure, especially in childhood, and laparotomy may assist the natural means which the body possesses to battle against the disease. The failure of a cure in early laparotomy is due to the fact that the operation was undertaken at a stage when the bacilli have not yet reached their complete virulence; operation done at a later stage when retrogressive changes have taken place in the life of the germs causing the disease is followed by cure.

How is it to be explained? We know that the congestive hyperæmia in the lungs due to severe cardiac disease hinders the development of tuberculosis, causing retrogression of diseased foci when present, or even complete cure.

Bien induced congestive hyperæmia as a treatment in suitable cases of tubercular conditions of the joints and tendons, with favorable results. From this analogy, the author believes that the venous hyperæmia, which is present for some days after the operation, plays an important role in the cure of the disease.

John Duncan, of Edinburgh, says: "I don't see how we can escape the conclusion, that more than one cause is required to account for the advantage of laparotomy, and that the most important are problably the relief of tension, the removal of inflating fluid, introduction of air, and the mechanical interference."

In summing up the treatment, I would be inclined in most cases in the early stages to try the medical and hygienic measures, especially if the symptoms were not urgent, as in Case 4. But I feel that in the ascitic sero-membranous, the fibrous (in the early stages) and in those cases caused by extension from the pelvis, surgery is the remedy, involving little risk and giving the strongest hope of cure. Even in the flat abdomen (retracted form), where the matting of intestines and extensive adhesions interfere with the circulation and nutrition of the parts, I think surgical intervention is justifiable.

